# Design and production of conjugate vaccines against *S*. *Paratyphi A* using an O-linked glycosylation system in vivo

**DOI:** 10.1038/s41541-017-0037-1

**Published:** 2018-02-05

**Authors:** Peng Sun, Chao Pan, Ming Zeng, Bo Liu, Haoyu Liang, Dongshu Wang, Xiankai Liu, Bin Wang, Yufei Lyu, Jun Wu, Li Zhu, Hengliang Wang

**Affiliations:** 1 State Key Laboratory of Pathogen and Biosecurity, Beijing Institute of Biotechnology, 20 Dongdajie Street, Fengtai Beijing, 100071 China; 2 National Institutes for Food and Drug Control, 2 Tiantanxili, Beijing, 100050 China

## Abstract

Enteric fever, mainly caused by *Salmonella enterica* serovar *Paratyphi A*, remains a common and serious infectious disease worldwide. As yet, there are no licensed vaccines against *S*. *Paratyphi A*. Biosynthesis of conjugate vaccines has become a promising approach against bacterial infection. However, the popular biosynthetic strategy using N-linked glycosylation systems does not recognize the specialized O-polysaccharide structure of *S*. *Paratyphi A*. Here, we describe an O-linked glycosylation approach, the only currently available glycosylation system suitable for an *S*. *Paratyphi A* conjugate vaccine. We successfully generated a recombinant *S*. *Paratyphi A* strain with a longer O-polysaccharide chain and transformed the O-linked glycosylation system into the strain. Thus, we avoided the need for construction of an O-polysaccharide expression vector. In vivo assays indicated that this conjugate vaccine could evoke IgG1 antibody to O-antigen of *S*. *Paratyphi A* strain CMCC 50973 and elicit bactericidal activity against *S*. *Paratyphi A* strain CMCC 50973 and five other epidemic strains. Furthermore, we replaced the peptides after the glycosylation site (Ser) with an antigenic peptide (P2). The results showed that the anti-lipopolysaccharide antibody titer, bactericidal activity of serum, and protective effect during animal challenge could be improved, indicating a potential strategy for further vaccine design. Our system provides an easier and more economical method for the production of *S*. *Paratyphi A* conjugate vaccines. Modification of the glycosylation site sequon provides a potential approach for the development of next-generation “precise conjugate vaccines.”

## Enteric fever: Developing a novel vaccine for a developing-world affliction

When bound to an immune system stimulator, bacterial surface molecules can elicit antibodies against the main cause of paratyphoid fever. Paratyphoid fever is highly similar to typhoid fever; however, without any licensed vaccine. Hengliang Wang, Li Zhu, Jun Wu and Ming Zeng, alongside a team of scientists from China’s Beijing Institute of Biotechnology and National Institutes for Food and Drug Control, developed a vaccine consisting of surface sugar chains from ‘*Salmonella enterica* serovar Paratyphi A.’ The chains were bound to a protein known to provoke the immune system (cholera toxin B subunit) and boost potential immunity. The vaccine generated protective antibodies against the *S. enterica* bacteria, was improved when the protein was altered to be more immune-reactive and offered a more practical, economic synthesis route compared to previous ‘conjugated’ vaccines.

## Introduction

Enteric fever, caused by several *Salmonella enterica* subspecies *enterica* serovars that are spread through contaminated water, food, flies, and cockroaches, remains an important cause of morbidity and mortality in developing countries, especially in Asia.^1^ In the past, *S*. Typhi was considered the major pathogenic serovar responsible for enteric fever. However, in recent years, *S*. *Paratyphi A* has gradually become the main cause of enteric fever because of the development of an efficient typhoid vaccine.^2–4^ In 2000, there were an estimated 5,412,744 cases of paratyphoid fever, mainly concentrated in the Indian subcontinent and South East Asia.^5^ In some areas, incidence rates are increasing annually.^6^ Paratyphoid fever is symptomatically indistinguishable from typhoid fever, and the development of resistance (such as that to nalidixic acid, chloramphenicol, ampicillin, and quinolones) has made treatment much more difficult.^7,8^

Vaccines have played an important role in the prevention of enteric fever. However, there is no vaccine licensed against *S*. *Paratyphi A*. At present, most prospective *S*. *Paratyphi A* vaccines are attenuated oral vaccines and glycoconjugate vaccines.^6^ Glycoconjugate vaccines contain a bacterial antigenic polysaccharide [O-polysaccharide (OPS) or capsular polysaccharide (CPS)] covalently attached to an appropriate carrier protein. This allows the polysaccharide antigen to be transformed from a T-cell-independent antigen into a T-cell-dependent antigen, thereby inducing humoral immunity and immune memory.^9–11^ Because of their ability to induce both T-cell-dependent and T-cell-independent immune responses, glycoconjugate vaccines are considered one of the most successful vaccine types.^12^ Various glycoconjugate vaccine against *S*. *Paratyphi A* are currently being examined, including O:2,12-TT + Vi-TT (phase II), O:2,12-CRM197 + Vi-CRM197 (preclinical), and O:2,12-DT + Vi-DT (preclinical).^6^ In all of these vaccines, chemical methods are required to extract and purify the polysaccharide and protein, respectively, and the final product is again purified following chemical cross-linking. These multiple steps are time-consuming and costly, which limits its marketing, especially in developing and poor countries where the demand for vaccines is the greatest.

Here, we describe a new method for producing a glycoconjugate vaccine against *S*. *Paratyphi A*. With the discovery of protein glycosylation in bacteria^13^ and the successful expression of an exogenous glycosylation system in *Escherichia coli*,^14,15^ a demonstrably superior bio-method, whereby the glycoconjugate vaccine is prepared in vivo, is becoming popular. In this method, bacterial surface polysaccharide antigens (OPS and some CPS), synthesized on undecaprenyl pyrophosphate (UndPP),^16^ are recognized by glycosyltransferase and transferred to carrier proteins containing a specific glycosylation site. Specific requirements for glycosylation sites and catalytic reactions in the periplasmic space largely prevent glycosylation of host proteins. Using this method, only three vectors (expressing the polysaccharide gene cluster, glycosyltransferase, and the carrier protein) need to be expressed in *E. coli*, and the glycoprotein is produced following a one-step purification method after induction.^12^ Compared with the earlier chemical methods, the bio-method is a low-cost, high-efficiency process, and the products are also more uniform.

A glycoconjugate vaccine against *Shigella dysenteriae*, produced in *E. coli* CLM24 using PglB, an N-linked glycosyltransferase from *Campylobacter jejuni*, has been developed by GlycoVaxyn AG, and phase I clinical trials have been completed.^17^ Unfortunately, the first sugar substrate of OPS in *S*. *Paratyphi A* does not contain an acetamido group,^18,19^ meaning that it cannot be transferred by PglB to produce a glycoconjugate vaccine.^20,21^ In our previous study, another O-linked glycosyltransferase, PglL from *Neisseria meningitides*, was used to produce a glycoconjugate vaccine.^22^ Compared with PglB, PglL has a more relaxed substrate specificity, and almost all types of glycan can be transferred.^23^ To glycosylate the carrier protein, a fragment (^45^SAVTEYYLNHGEWPGNNTSAGVATSSEIK^73^) in which Ser63 was the glycosylation site was selected from the sequence of PilE, the natural substrate of PglL,^24,25^ and fused to the C-terminus of the cholera toxin B subunit (CTB), resulting in the fusion protein named CTB4573. At the same time, a preliminary study showed that a conjugate vaccine could be prepared directly in the attenuated pathogenic bacterium,^22^ which avoided synthesis and expression of the O-antigen gene cluster and simplified the vaccine preparation process.

In the current study, we replaced the polysaccharide chain length regulator gene *cld* with its counterpart from *S. typhimurium*, resulting in a longer polysaccharide chain, which was used to produce an *S*. *Paratyphi A* conjugate vaccine using the bio-method. Our results showed that the glycoprotein could evoke a protective and specific immune response. We also explored a new strategy to enhance vaccine immunogenicity by modifying the glycosylation sequon. Such modifications evoked a better immune response, providing a method to further study next-generation glycoconjugate vaccines.

## Results

### Construction of an O-linked glycosylation system in *S*. *Paratyphi A* with a longer OPS structure

Our previous work showed that co-expression of O-linked glycosyltransferase PglL and CTB4573H (6×His-Tag fused at the C-terminal of CTB4573) resulted in glycosylation of carrier protein CTB4573H in strain 50973DW, an O-antigen ligase gene *waaL* knockout strain of *S*. *Paratyphi A* strain CMCC 50973 (SPA50973) (Supplementary Fig. 1). These results indicated that a bioconjugate vaccine for *S*. *Paratyphi A* could be produced using this O-linked glycosylation system. However, previous studies reported that the use of short polysaccharide chains, such as the OPS of SPA50973, in conjugate vaccines may not induce a sufficient immune response.^26–29^ In addition, the short chain makes purification and separation of glycosylated and unglycosylated proteins more difficult.

To solve the problem of the short chain length of the OPS in SPA50973, we first knocked out the native *cld* (controlled the length of the polysaccharide chain) using the λ Red recombination system (Supplementary Fig. 2A). The *S. typhimurium* *cld* gene, *cld*_LT2_, which is regulated to produce a long polysaccharide chain, was then cloned into vector pACU184 under the control of the Tac promoter, generating plasmid pACU184-cld_LT2_. The recombinant plasmid was transformed into the *cld* knockout strain, resulting in strain 50973DC/Cld_LT2_. Following induction with isopropyl-β-D-thiogalactopyranoside (IPTG), the lipopolysaccharide (LPS) of each strain was analyzed by sodium dodecyl sulfate polyacrylamide gel electrophoresis (SDS–PAGE) and silver staining. The results showed the typical extended ladder for the IPTG-induced strain expressing Cld_LT2_, and the number of repeat units was >20 (Supplementary Fig. 2B). This indicated that Cld_LT2_ could control the length of OPS chains in *S*. *Paratyphi A*. To examine glycosylation, we constructed strain 50973DWC, containing mutations in both *waaL* and *cld*, as described above. Western blot analysis showed that when recombinant plasmid pET28a-pglL-CTB4573H (expressing inducible PglL and CTB4573H) was transformed into strains 50973DW and 50973DWC, glycosylated carrier protein CTB4573H was present in both strains following IPTG induction. However, bands corresponding to glycosylated CTB4573H in strain 50973DWC were of a lower molecular weight than those in strain 50973DW (Supplementary Fig. 3), indicating the loss of function of Cld in 50973DWC.

**Fig. 1:**
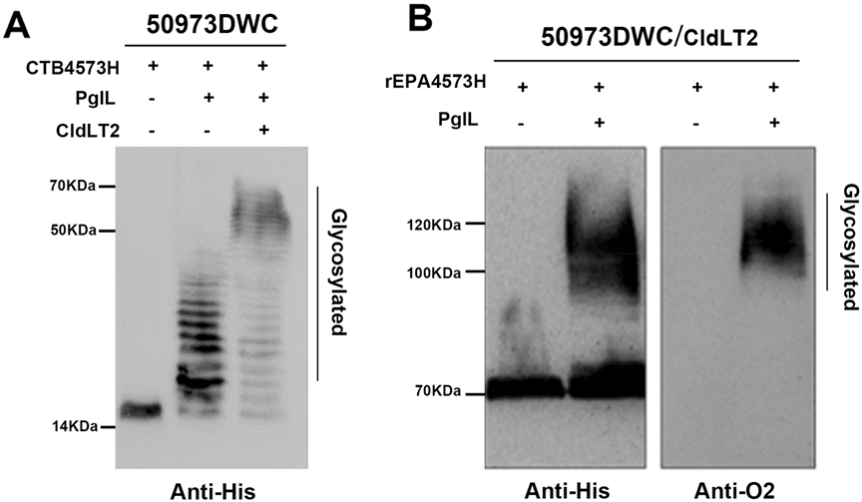
Glycosylation of the extended polysaccharide chain. **a** A plasmid co-expressing CTB4573H and PglL was transformed into *S*. *Paratyphi A* *waaL*/*cld* double mutant strain 50973DWC. Western blot assays were conducted to analyze glycosylation of CTB4573H with or without Cld_LT2_ in 50973DWC. Samples were derived from the same experiment. **b** Western blot analysis of glycosylated rEPA4573H with the extended polysaccharide chain co-expressing Cld_LT2_, rEPA4573H, and PglL. Samples were derived from the same experiment and gels/blots were processed in parallel

To verify that the longer OPS chain could be transferred to the carrier proteins, CTB and a widely used commercial carrier protein, recombinant *Pseudomonas aeruginosa* exotoxin A (rEPA), were used. Plasmids pET28a-pglL-CTB4573H and pET28a-pglL-rEPA4573H were introduced separately into strain 50973DWC carrying plasmid pACU184-cld_LT2_. Western blot analysis showed high molecular weight bands corresponding to glycosylated CTB4573H and rEPA4573H (~60 kDa and 100 kDa, respectively) were only present in strains co-expressing CTB4573H/rEPA4573H, PglL, and Cld_LT2_ (Fig. 1a, b).

### Purification and analysis of glycoprotein

**Fig. 2:**
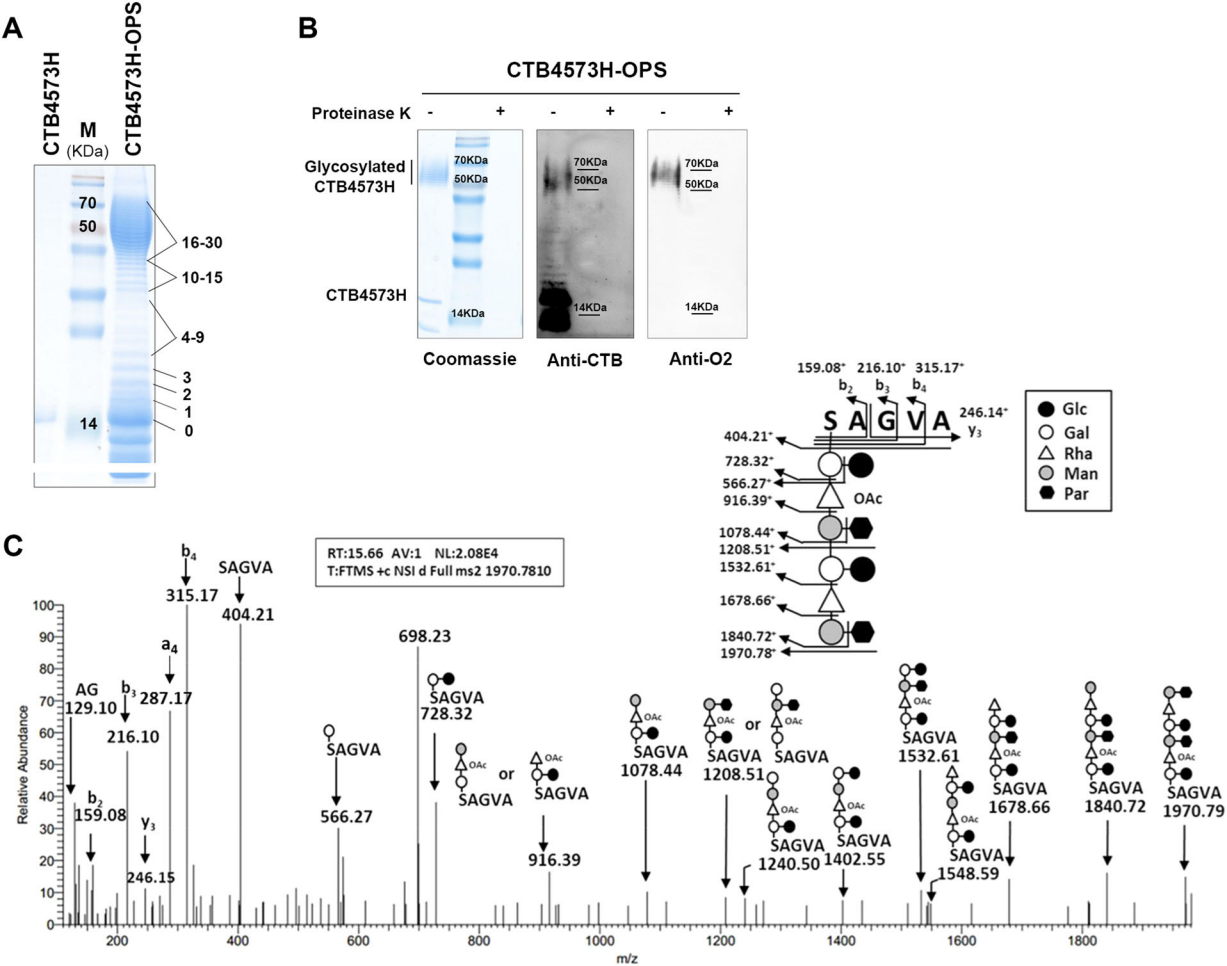
Analysis of glycosylation of CTB4573H with extended O-antigen. **a** Coomassie blue staining of purified CTB4573H-OPS to analyze the number of O-antigen repeat units. Samples derived from the same experiment and were processed in parallel. **b** Coomassie blue staining and western blot assays were conducted to detect glycosylated CTB4573H incubated with or without proteinase K using anti-CTB antibody and *S*. *Paratyphi A* O2 serum. Samples were derived from the same experiment and gels/blots were processed in parallel. **c** Tandem mass spectrometry (MS/MS) analysis of glycosylated CTB4573H from *S*. *Paratyphi A* OPS. MS/MS spectrum of singly charged ions at *m/z* 1970.78^+^ contained the pentapeptide SAGVA (*m/z* 404.21^+^) and a peptide linked with sugars. The peptide and glycopeptide fragment ions were identified (shown in the inset)

Glycosylated CTB4573H (CTB4573H-OPS) and rEPA4573H (rEPA4573H-OPS) were purified from 50973DWC strains co-expressing Cld_LT2_, PglL, and CTB4573H/rEPA4573H by affinity chromatography. The structural characteristics of the glycoprotein were analyzed from purified CTB4573H-OPS. Coomassie blue staining showed that Cld_LT2_ regulated the glycan in CTB4573H-OPS, with an average modal length of ~16–30 repeat units (Fig. 2a). To confirm the specificity of OPS, anti-O2 serum specific for *S*. *Paratyphi A*, along with CTB antibody, was used to detect glycan subunits of the glycoproteins with and without proteinase K treatment. Western blot analysis showed the presence of characteristic bands in samples not treated with proteinase K, while no bands were observed in proteinase K-digested samples, indicating that the purified sample did not contain LPS residues (Fig. 2b). Size-exclusion high-performance liquid chromatography (SEC-HPLC) confirmed that the purity of CTB4573H-OPS was 94%, which was suitable for further analyses (Supplementary Fig. 4).

To determine the structure of the glycans, the band of CTB4573H-OPS was excised from the SDS–PAGE gel. Following proteinase K digestion, the glycopeptide was examined by liquid chromatography tandem-mass spectrometry (LC-MS/MS), and the structure was analyzed based on the known structure of *S*. *Paratyphi A* OPS^18,19^ (Fig. 2c). In the two repeat units, only one O-acetylation at the C-3 position of rhamnose was detected, consistent with previous studies that O-acetylation did not occur in each repeat unit.^19^ Coomassie blue staining and western blot analysis (anti-*S*. *Paratyphi A* O2 serum and EPA antibody) of the purified rEPA4573H-OPS also showed high purity (Supplementary Fig. 5).

### Evaluation of immune effects in mice

Purified CTB4573H-OPS, rEPA4573H-OPS, and OPS, along with 10% Al(OH)_3_ adjuvant (General Chemical), were used to immunize 5-week-old BALB/c mice. Each group of ten animals was inoculated subcutaneously with the same amount of polysaccharides (2.5 µg/mouse) or adjuvant alone on days 1, 15, and 29. Serum was collected pre-immunization and 10 days after each immunization, and an enzyme-linked immunosorbent assay (ELISA) was performed to evaluate the induction of IgG antibody against SPA50973 LPS. Both CTB4573H-OPS and rEPA4573H-OPS could induce a stronger immune response in BALB/c mice than OPS alone, however, the average titer of the CTB4573H-OPS group was significantly higher (*P* < 0.001 after the third immunization) than that of the rEPA4573H-OPS group (Supplementary Fig. 6). These results indicated that at this dose, CTB had greater potential as a carrier than rEPA, which may be related to the pentameric form of glycosylated CTB (Supplementary Fig. 7). Hence, CTB was used for subsequent assays.

**Fig. 3:**
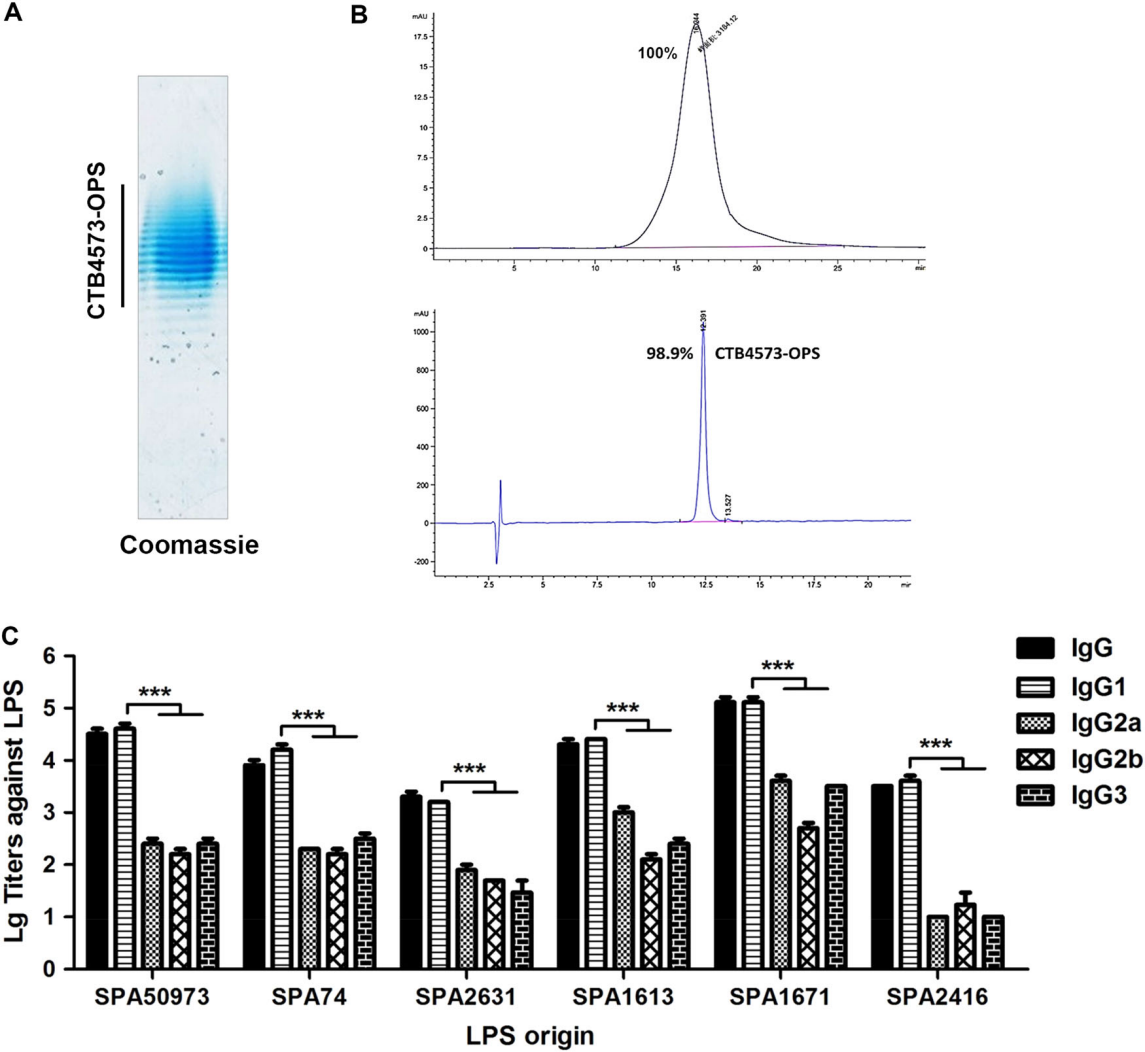
Analysis and immune effect evaluation of CTB4573-OPS in mice. **a** Coomassie blue staining of purified CTB4573-OPS. **b** Analysis of purified CTB4573-OPS by size-exclusion high-performance liquid chromatography (TSK G4000SWXL, diameter, 7.8 × 300 mm) (up) and reversed-phase high-performance liquid chromatography (Agilent ZORBAX 300SB-C8, diameter, 4.6 × 250 mm) (down). **c** IgG subclass titers (IgG1, IgG2a, IgG2b, and IgG3) against LPSs from six *S*. *Paratyphi A* strains were measured in serum from CTB4573-OPS immunized mice. A *t*-test was used to evaluate differences between IgG1 titer and other subclass titers (***P < 0.001). Error bars indicate the standard deviation

For commercial application, we constructed a plasmid, pET-pglL-CTB4573, to remove the 6×His-tag. CTB4573-OPS was expressed in 50973DWC strains co-expressing Cld_LT2_, PglL, and CTB4573. Purification was performed by ion exchange. The product was detected by Coomassie blue staining, showing a high purity (Fig. 3a). Approximately 10 mg (glycan component) of high-purity CTB4573-OPS could be obtained per liter of culture broth. The purity was nearly 100 and 98.9% by SEC-HPLC and reversed-phase high-performance liquid chromatography, respectively. Protein and glycan contents were measured by bicinchoninic acid (BCA) assays (Micro BCA Protein Assay Kit; Thermo Scientific) and the anthrone–sulfuric acid method,^22^ and the quantity ratio of saccharide/protein in CTB4573-OPS was 0.86:1. Following digestion with trypsin,^30^ three protein impurities were detected by LC-MS/MS (Supplementary Fig. 8A). Since glycosylated CTB could form a pentamer, we further evaluated the thermal stability of CTB4573-OPS polymer at different pHs (3.5–10.0) using the Protein Thermal Shift Dye Kit (Applied Biosystems). The melting temperature of the pentamer reached 62 °C in a wide pH range (pH 4.5–10.0) (Supplementary Fig. 8B). In addition, vaccination was performed subcutaneously on days 1, 15, and 29, with adjuvant only as the control. Serum was collected on day 39 (10 days after the last immunization) and ELISA results showed a high IgG antibody titer in the immunized serum against SPA50973 LPS (Supplementary Fig. 9A). To confirm whether these antibodies are protective against other *S*. *Paratyphi A* strains, sera of 60 mice were pooled together. Total IgG and IgG subclass titers (IgG1, IgG2a, IgG2b, and IgG3) against LPSs of SPA50973 and five other Chinese *S*. *Paratyphi A* epidemic strains (SPA74, SPA2631, SPA1613, SPA1671, and SPA2416) were detected. In all groups, IgG1 titers were significantly higher than those of other IgG subclasses (*P* < 0.001) (Fig. 3b). In addition, we measured the bactericidal activity of serum in vitro. The six *S*. *Paratyphi A* strains were incubated with different dilutions of serum (complement present in the immune serum was inactivated by incubation at 56 °C for 30 min), and then complement was added to the mixture. Bactericidal activity against all six strains could be induced by the antibody against SPA50973 OPS, and the activity for each strain coincided with the results of IgG assays (Supplementary Fig. 9B). The results suggested a correlation between low binding of the antibody to LPS and poor bactericidal activity.

### Modification of the glycosylation site sequon and evaluation of the immune effect

**Fig. 4:**
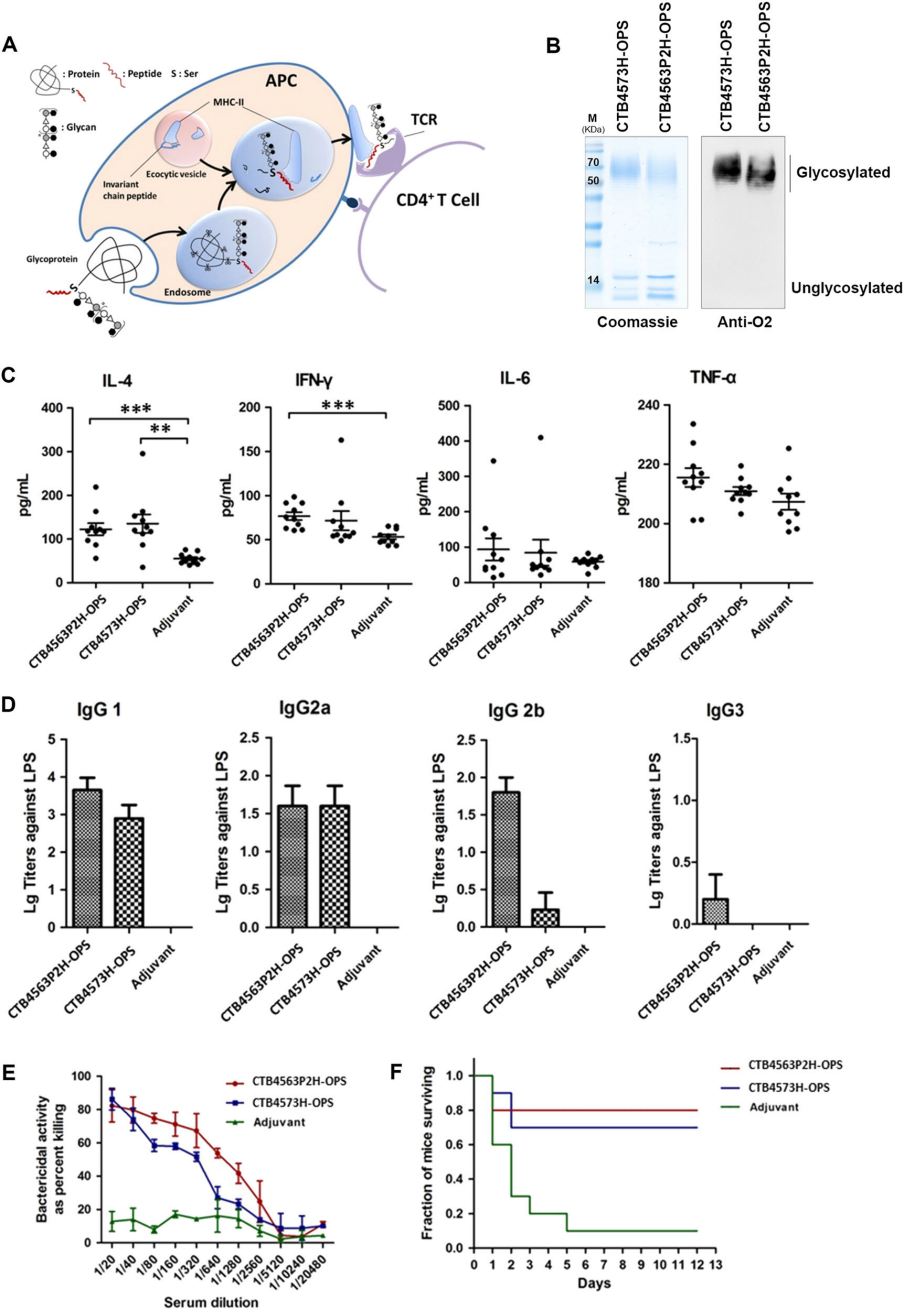
Evaluation of the immune effect of the glycoprotein with different antigen peptides. **a** Model of glycogen-specific antigen presentation to T-cells. The glycoprotein is taken up by the antigen-presenting cell (APC) and digested into multiple peptides and glycan-peptides in the endosome. The peptide (red) component of the glycan-peptide is then recognized and bound by MHC-II. Finally, the glycan-peptide-MHC-II complex is transferred to the surface of the APC where it is recognized by T-cell receptors (TCRs), evoking T-cell-dependent immune responses. **b** The purified CTB4573H-OPS and CTB4563P2H-OPS glycoproteins were detected by Coomassie blue staining and western blot analysis against *S*. *Paratyphi A* O2 serum. Samples were derived from the same experiment and gels/blots were processed in parallel. **c** IL-4, IL-6, TNF-α, and IFN-γ levels were measured in the serum collected after the last immunization with CTB457H-OPS, CTB4563P2H-OPS, or adjuvant alone by ELISA (mouse IL-4, IL-6, TNF-α, and IFN-γ ELISA kit; Dakewe). A *t*-test was used to compare the results from the treatment groups with the expression levels of the control group (**P < 0.01; ***P < 0.001). **d** IgG subclass titers (IgG1, IgG2a, IgG2b, and IgG3) against SPA50973 LPS were measured in serum as described for panel **c**. **e** Complement bactericidal activity was measured in different dilutions of serum as described for panel **c**. Error bars indicate the standard deviation. **f** Two weeks after final immunization with each conjugate vaccine, mice were infected intraperitoneally with ~5.76 × 10^7^ CFU/mouse of SPA50973 and the survival was monitored

According to the antigen presentation process (Fig. 4a), glycoprotein is taken up by antigen-presenting cells (APCs) and hydrolyzed into short peptides in the endosome. The glycan portion can only be presented by major histocompatibility complex class II (MHC-II) when linked to an MHC-II-binding peptide on the carrier protein. The glycan–peptide–MHC-II complex can then be transferred to the T-cell receptor (TCR), evoking humoral immunity (11, 25). Therefore, the binding affinity between the glycosylated peptide and MHC-II influences the efficacy of the immune response. We hypothesized that the immunogenicity of conjugate vaccines could be improved by increasing the presentation efficiency of the peptide-binding glycan. To examine this hypothesis, we modified the natural glycosylation site peptide sequence (4573) in CTB according to a previous analysis on 4573.^22^ Briefly, the two amino acids following glycosylation site Ser63 were changed to AP, and then fused to a P2 sequon (TT^830–843^: QYIKANSKFIGITE), which is a helper T-cell epitope derived from tetanus toxoid (TT) that may have strong binding capacity for MHC-II.^31,32^ We subsequently obtained a new glycosylation sequon (4563P2: SAVTEYYLNHGEWPGNNT**S**APQYIKANSKFIGITE), which differs from the natural sequon (4573: SAVTEYYLNHGEWPGNNT**S**AGVATSSEIK). This recombinant vector was named pET28a-pglL-CTB4563P2H and transformed into strain 50973DWC carrying pACU184-cld_LT2_. Following IPTG induction, western blotting was performed, which showed that long OPS chains could bind to CTB4563P2H in strains co-expressing Cld_LT2_, PglL, and CTB4563P2H (Supplementary Fig. 10). The amount of glycoprotein CTB4563P2H-OPS did not appear to be reduced compared with CTB4573H-OPS. Purification was performed as described above, and the resulting protein was examined by western blotting and Coomassie blue staining (Fig. 4b).

To compare the effects of the different antigenic peptides, CTB4573H-OPS and CTB4563P2H-OPS were used to immunize BALB/c mice as described above. Examination cytokine concentrations (IL-4, IL-6, TNF-α, and IFN-γ) in the serum of immunized animals showed that IL-4 was increased in both CTB4573H and CTB4563P2H groups compared with the control group (Fig. 4c). ELISA assays were also carried out to further clarify IgG subclasses (IgG1, IgG2a, IgG2b, and IgG3) in the serum raised in response to SPA50973 LPS. Both glycoproteins stimulated an IgG1-based response and the IgG1 titer of the CTB4563P2H-OPS group was higher than that of the CTB4573H-OPS group (Fig. 4d). Moreover, the serum from CTB4563P2H-OPS-inoculated animals induced a higher level of complement bactericidal activity than that of CTB4573H-OPS-inoculated animals at each of the dilutions tested (Fig. 4e). To further evaluate the protective efficacy of the vaccines, immunized mice were administered 5.76 × 10^7^ CFU/mouse of *S*. *Paratyphi A* strain CMCC 50973 by intraperitoneal injection on day 43 (14 days after the last vaccination or control injection). We observed that deaths mainly occurred in the first few days, and animals from both CTB4563P2H-OPS and CTB4573H-OPS immunized groups high survival rates (Fig. 4f).

## Discussion

In this study, we produced an *S*. *Paratyphi A* conjugate candidate vaccine with an extended polysaccharide chain by establishing an O-linked glycosylation system in *S*. *Paratyphi A* and evaluated its immunogenicity using two different carrier proteins and protective effects against different *S*. *Paratyphi A* epidemic strains. Based on the immune mechanism, we then developed a new modification strategy by altering the glycosylation sequon to make the vaccine more effective. In vivo experiments showed that the *S*. *Paratyphi A* conjugate candidate vaccine, produced using this bio-method, induced high-level protection against pathogenic bacteria, and optimization of the glycosylation sequon can improve vaccine immunogenicity.

In our system, the glycoprotein could be directly synthesized in vivo, and a different purification method, as opposed to chemical methods, was needed. We purified our product using the method employed for glycoprotein purification and the difference between general glycoprotein and our vaccine was sugar content, which was higher in our product. Thus, the characteristics of sugar should be considered during purification. At present, two glycosyltransferases (PglL and PglB) can be used in the development of polysaccharide conjugate vaccines. Although *C. jejuni* PglB (N-linked) was the first glycosyltransferase to be used to produce conjugate vaccines, it only recognizes glycans with an acetylated sugar at the reducing end and without a β1–4 linkage between the first two sugars.^20,21^ This limitation meant that conjugate vaccines could not be developed for bacteria such as *Streptococcus pneumoniae, Streptococcus suis*, and *S*. *Paratyphi A*.^33–36^ Therefore, we developed another system based on the PglL glycosyltransferase from *Neisseria meningitides*, which recognizes almost all polysaccharides.^23^ In addition, some group I and IV CPS, synthesized on UndPP, can theoretically be identified by PglL. In this new system, *cld*_LT2_ from *S. typhimurium* was used to extend the chain length, as the OPS from *S*. *Paratyphi A* and *S*. *typhimurium* share the same trisaccharide backbone. However, sharing an identical backbone may not be essential for successful expression, as demonstrated by another study which aimed to lengthen the OPS of *Vibrio cholerae* O139.^37^ The main difference in the OPS structure between *S*. Paratyphi serogroup A and serogroup B and D strains is the presence of a single a-3,6-dideoxyglucose (a-D-paratose), located at the C-3 position of mannose in serogroup A strains. This additional sugar plays a decisive role in the specificity of OPS^19^ and could be detected by O2 serum in western blot analyses. The extended polysaccharide chain of the conjugate vaccine developed in the current study did not affect antigen specificity, as demonstrated by analysis of antibody levels against natural LPS, bactericidal activity, and protective effect of the vaccine in mice against *S*. *Paratyphi A*. Thus, control of the polysaccharide chain length may be achieved through the introduction of different *wzz*/*cld* genes, providing a method to optimize OPS, especially in species such as *V. cholerae* O139, which has only one repeat unit.

One advantage of the bio-method vaccine design is its versatility because bioconjugate vaccines are developed through molecular biological methods. In the immune process, the peptide-bonded glycan can be likened to a carrier vehicle, which is responsible for the transportation of saccharide from APC to TCR. The finding that amino acids at the C-terminal of the glycosylation site (S) are not essential suggested that the peptide segment immediately following S could be altered.^22^ Many epitopes have been described, such as hemagglutinin peptide residues 307–319 (HA^307–319^), P2, and tetanus peptide residues 947–967 (P30), among others.^31,32,38,39^ Several of these epitopes have been used for vaccine design, including P2 and P30, to great effect.^31,32^ In the current study, we chose peptide fragment P2 to fuse onto the C-terminal of glycosylation site S with a two-amino acid linker, AP. In vivo experiments showed that serum antibody titers and bactericidal ability of the P2 fusion were increased. However, only two polypeptides, 4573 and 4563P2, were compared in this study. In the future, we plan to select a variety of peptides with antigen-presenting ability to generate a range of glycoproteins, which will then be examined for their immunogenicity. Based on the design of the vaccine production method developed in this study, we named this type of vaccine a “precise conjugate vaccine.” As such, another advantage of the bio-method is that the glycoconjugate vaccine can be precisely designed by modification of the peptide around the glycosylation site, Ser.

In the current study, serum IL-4 levels in vaccinated animals were significantly higher than that in the control. IL-4 plays an important role in humoral immunity by inducing the differentiation of Th0 cells into Th2 cells and mediating the production of IgG1 antibodies, which may activate the classical complement pathway and provide long-term protection. Furthermore, IL-4 is known to antagonize Th1 cell differentiation. According to the ELISA results of the current study, which showed the greatest induction of IgG1 by LPS, humoral immunity was the main response. In contrast, polysaccharide-only vaccines can stimulate cellular immunity, whereby IgG2a is the main antibody induced.

Overall, the results of this study confirm that the O-linked glycosylation system is useful for preparation of an *S*. *Paratyphi A* conjugate vaccine. The strategy of preparing the vaccine directly in recombinant *S*. *Paratyphi A* was easier than methods using *E. coli* because it avoided cloning of the polysaccharide synthesis gene cluster, a huge fragment of >10 kb. The O-linked glycosylation system as a platform could easily produce conjugate vaccines for a number of bacteria, including *V. cholerae, Acinetobacter baumannii*, and *S. pneumoniae*. Importantly, this method allows the precise design of the polysaccharide conjugate vaccine through modification of the glycosylation site sequon. This is a unique advantage of the bio-method and can improve the immunogenicity of bioconjugate vaccines. Overall, this method paves the way for further research and development of conjugate vaccines.

## Methods

### Strains, growth conditions, and plasmids

50973DW, a *waaL* mutant of *S*. *Paratyphi A* strain CMCC 50973, was described in our previous work.^22^ Chinese *S*. *Paratyphi A* epidemic strains (SPA74, SPA2631, SPA1613, SPA1671, and SPA2416) were provided by the Institute for Communicable Disease Control and Prevention, Chinese Center for Disease Control and Prevention, Beijing, China. All bacterial strains were cultured in Luria–Bertani (LB) broth or on solid LB medium containing 1.5% agar. *E. coli* DH5α (TransGen Biotech) was used for cloning experiments and cultured at 37 °C. For the induction of glycosylation, cells were first cultured in LB at 37 °C to an optical density at 600 nm (OD_600_) of approximately 0.5. Then, 1 mM IPTG was added to induce protein expression at 30 °C for 10 h. When needed, kanamycin and chloramphenicol (both at 50 μg/mL) were added to culture medium. The plasmids used in this study are listed in Table 1.

**Table 1:** Main plasmids used in this study

Plasmids	Characteristic	Source
pKD3	Template plasmid for chloramphenicol resistance fragment flanked by FRT (FLP recognition target) sites	Laboratory stock
pKD46	used for λ-red recombination, araC-ParaB, ApR	Laboratory stock
pCP20	Used for the removal of chloramphenicol gene	Laboratory stock
pACU184-cld_LT2_	Encodes Cld from *S. typhimurium* and under control of Tac promoter, Cm^R^	This work
pET-pglL-rEPA4573H	Encodes PglL and 6×His-tagged rEPA4573, both of them are under control of Tac promoter, Kan^R^	Laboratory stock
pET-pglL-CTB4573H	Encodes PglL and 6×His-tagged CTB4573, both of them are under control of Tac promoter, Kan^R^	Laboratory stock
pET-pglL-CTB4573	Similar with pET-pglL-CTB4573 but have no 6×His-tag	This work
pET-pglL-CTB4563P2H	Encodes PglL and 6×His-tagged CTB4563P2, both of them are under control of Tac promoter, Kan^R^	This work

### Construction of an *S*. *Paratyphi A* strain CMCC 50973 *waaL* and *cld* double mutant strain

An SAP50973 *waaL*/*cld* double knockout strain was constructed using the λ Red recombination system as described previously.^40^ The double mutant, named 50973DWC, was generated using an SPA50973 Δ*waaL* strain (50973DW) previously constructed in our laboratory.^22^ Briefly, two ~500-bp fragments corresponding to the regions immediately adjacent to *cld* were amplified from SPA50973 genomic DNA using upstream and downstream primers cld-UP5 and cld-UP3 (5′-CAGTTGCTGGCTAATTATCAGTCAGTGCCT-3′ and 5′-GTCATAGATACCCTAACTAAAAAAAGGATGAAGC-3′) and cld-DOWN5 and cld-DOWN3 (5′-TTCTTTGCCGGATGGTGGTCGGCTTCGAAA-3′ and 5′-TTATGGACCAAAGGCGAAACCTCAGGCCAT-3′). A chloramphenicol resistance fragment flanked by FLP recognition target sites and a 41-bp homologous sequence was amplified from pKD3 using primers cat-5 and cat-3 (5′-CCAGCTTCATCCTTTTTTTAGTTAGGGTATCTATGACAAGCGATTGTGTAGGCTGGAG-3′ and 5′-CCTTTCGAAGCCGACCACCATCCGGCAAAGAAGCTAATTAACGGCTGACATGGGAATTAG-3′). The three fragments were connected by overlap PCR using primers cld-UP5 and cld-DOWN3, and the products were introduced into SPA50973DW/pKD46 competent cells by electroporation. Following overnight culture at 30 °C, colonies containing the correct construct were identified by PCR using primers cld-UP5/cld-DOWN3. Plasmid pCP20 was then introduced to excise the antibiotic gene, and temperature-sensitive plasmids pKD46 and pCP20 were cured by incubating the culture at 42 °C.

### Western blot analysis

Western blotting was performed as described previously.^41^ Horseradish peroxidase (HRP)-conjugated 6×His-tag antibody (Abmart) was used to detect His-tag-fused proteins, anti-EPA antibody from rabbit (Sigma) (1:7,500) was used to detect rEPA, anti-CTB antibody from rabbit (Abcam) (1:200) was used to detect CTB, and antiserum (Denka Seiken) (1:200) specific for *S*. *Paratyphi A* OPS, produced in rabbits, was used to detect the polysaccharide of glycoproteins.

### Glycoprotein purification

*S*. *Paratyphi A* cells were pelleted by centrifugation at 10,000×*g* for 15 min at 4 °C and then resuspended in buffer A1 [20 mM Tris-HCl (pH 7.5), 0.5 M NaCl, 10 mM imidazole]. Cells were then homogenized and centrifuged at 14,300×*g* for 20 min at 4 °C (Beckman Instruments, Inc.). The supernatant was applied to a chelating column (5.0 × 15 cm diameter, GE Healthcare). After washing with buffer A1, bound protein was eluted in 50% buffer B1 [20 mM Tris-HCl (pH 7.5), 0.5 M NaCl, 0.25 M imidazole] and desalted on a G25 fine column (5.0 × 50 cm diameter, GE Healthcare) with buffer A2 (20 mM HAc-NaAc, pH 5.5). The fraction containing glycoprotein was applied to a Source 30S column (2.5 × 12 cm diameter, GE Healthcare) and washed with buffer A2. The bound glycoprotein was eluted using a stepwise gradient of 25%, 45%, and 100% buffer B2 [20 mM HAc-NaAc (pH 5.5), 1 M NaCl]. Fractions containing glycoprotein were collected and analyzed by 12% SDS–PAGE.

### In-gel protein digestion

Glycosylation bands were excised from the SDS–PAGE gel following Coomassie blue staining. Gel slices were incubated with 50% (v/v) acetonitrile in 50 mM ammonium bicarbonate three times to remove all dye. Gel slices were then dried by SpeedVac, and proteins were reduced by the addition of 10 mM DTT in 50 mM ammonium bicarbonate solution. The slices were washed twice with 10 mM ammonium bicarbonate followed by 100% acetonitrile and dried by SpeedVac. Proteinase K (50 μg/mL) was added to gel slices to digest the proteins, and samples were incubated at 56 °C for 3 h. An equal volume of 60% (v/v) acetonitrile in 5% (v/v) formic acid was added, and the supernatant was obtained by pipetting. This step was repeated, and then the peptides contained in the resulting supernatant were dried by SpeedVac and finally dissolved in 0.1% (v/v) formic acid.

### Mass spectrometry

A 2-μL aliquot of the peptide solution was separated on a C18 nano column (25 cm) using the EASY-nLC system (Thermo Fisher Scientific). Samples were then loaded and eluted for 120 min using a 4–90% acetonitrile fraction-optimized nonlinear gradient in 0.1% formic acid. Eluted peptides were detected on an Orbitrap Fusion Lumos Tribrid mass spectrometer (Thermo Fisher Scientific).

### Animals and immunization experiments

Specific-pathogen-free mice were purchased from the Laboratory Animal Center of the Academy of Military Medical Sciences, Beijing, China, and housed with a constant ambient temperature (23 ± 3 °C) and humidity (55 ± 5%). All animal experiments were approved by and performed in accordance with the recommendations of the Academy of Military Medical Sciences Institutional Animal Care and Use Committee. Female BALB/c mice (5 weeks old) were used in immunization experiments and randomly divided into groups, with ten animals each. The size of the group chosen was to ensure the adequate power to obtain statistically relevant data. These groups were not blinded to the investigators. Purified glycoproteins were diluted in phosphate-buffered saline (PBS) and mixed with 10% aluminum hydroxide adjuvant (General Chemical). Mice without any treatment (blank group) were used as a control. Immunizations were performed subcutaneously on days 1, 15, and 29, with the control group receiving adjuvant only. On day 39 (10 days after the last immunization), blood samples were taken by tail snip, incubated at 37 °C for 2 h, and then the serum was collected and stored at 4 °C until further analysis.

### LPS preparation

Strain was cultured in LB medium at 37 °C for 12 h. The culture was then centrifuged at 7000×*g* for 10 min at 4 °C, and the cell pellet was washed three times with ddH_2_O. Washed cells were resuspended in 3 mL of ddH_2_O per gram of cells. After freeze-thawing the samples three times, an equal volume of 90% phenol was added, and samples were shaken vigorously for 30 min at 68 °C. The mixture was then centrifuged at 7000×*g* for 20 min at 4 °C, the uppermost part of the solution was collected, and the phenol layer was re-extracted with an equal volume of ddH_2_O. The two extracts were then combined and dialyzed using a dialysis bag (MW 3500) in ddH_2_O for 2 days. DNase (5 µg/mL; Amresco) and RNase (1 μg/mL; Amresco) were added to dialyzed samples, which were then incubated at 37 °C for 4 h. Samples were then treated with proteinase K (10 μg/mL; Merck) at 60 °C for 1 h. After 10 min in a boiling water bath, samples were centrifuged at 40,000×*g* for 20 min to obtain LPS.

### ELISA

LPS, diluted in carbonate coating buffer (50 mM Na_2_CO_3_–NaHCO_3_, pH 9.6) to 100 μg/mL, was used to coat 96-well immunoplates, which were incubated at 4 °C overnight. The wells were then washed with wash buffer [PBS supplemented with 0.05% (v/v) Tween 20] using a plate washer (BioTek Instruments, Inc.). After being patted dry, 200 μL of blocking buffer containing 5% milk in PBS were added to each well, and plates were then incubated for 2 h at 37 °C. Plates were again washed and patted dry. Immune serum was serially diluted in dilution buffer [PBS supplemented with 0.5% (w/v) milk], and 100 μL of each dilution were added to the wells of the 96-well plate and incubated for 1 h at 37 °C. After another washing and drying step, 100 μL of one of the following HRP-conjugated goat anti-mouse antibodies (Abcam), diluted 1:20,000 in dilution buffer, were added to the wells of the 96-well plate and incubated for 1 h at 37 °C: IgG, IgG1, IgG2a, IgG2b, or IgG3. Plates were again washed and dried, and then 100 μL of color solution (OPD-H_2_O_2_ solution) were added to each well, and the plates were incubated for 15 min at room temperature. The reaction was halted by adding 50 μL of stop solution (2 mol/L H_2_SO_4_). A microplate spectrophotometer was used to measure the absorbance at a wavelength of 490 nm.

### Complement sterilization experiments

*S*. *Paratyphi A* cells were cultured at 37 °C to an OD_600_ of approximately 2.0, corresponding to ~1.2 × 10^9^ CFU/mL, and then diluted to ~2.0 × 10^4^ CFU/mL in normal saline. Immune sera were pools that contained equal volumes of serum samples from ten mice in each group, and complement was inactivated by incubation at 56 °C for 30 min. A 10-μL aliquot of the inactivated immune sera was diluted to varying concentrations, with normal saline as a control. Then, 10 μL of diluted *S*. *Paratyphi A* culture were added to each dilution. Following incubation for 1 h at room temperature, 20 μL of complement (Pel-Freez) (provided by Lanzhou Institute of Biological Products Co., Ltd, Lanzhou, China) were added and the mixtures were incubated at 37 °C for 1 h. Following incubation, the mixtures were plated on LB agar medium and incubated overnight at 37 °C. The resulting CFU for each sample was used to calculate the percentage of surviving cells.

### Statistical analyses

Antibody titers were log10-transformed and relevant statistical tests are indicated in figure legends and were conducted using GraphPad Prism version 5.0 and SAS 9.2. Differences were considered statistically significant at *P < *0.05.

### Data availability

All relevant data from this study are available from the authors.

## Electronic supplementary material

Full length gels-blots

Supplemental figure legends

Supplementary Fig. 1

Supplementary Fig. 2

Supplementary Fig. 3

Supplementary Fig. 4

Supplementary Fig. 5

Supplementary Fig. 6

Supplementary Fig. 7

Supplementary Fig. 8

Supplementary Fig. 9

Supplementary Fig. 10
