# Supplementary material for: Design and production of conjugate vaccines against S. Paratyphi A using an O-linked glycosylation system in vivo
Source: NPJ Vaccines. 2018 Feb 5;3:4. doi: 10.1038/s41541-017-0037-1 (PMC5799188; doi:10.1038/s41541-017-0037-1)

**Full length gels/blots for each gel blot figure:**

**Figure 1.**

**A.**


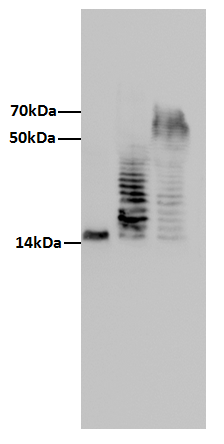


**B.**


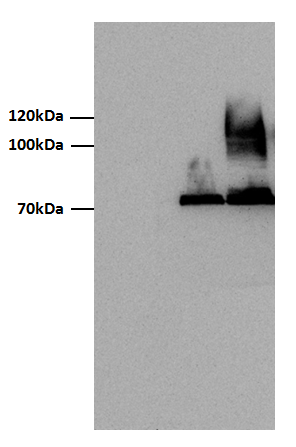

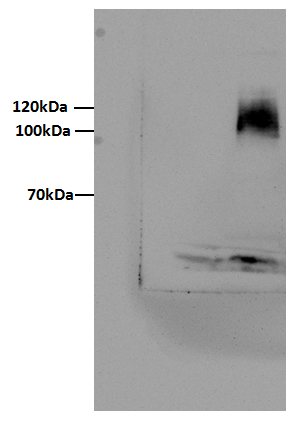


**Figure 2.**

**A.**


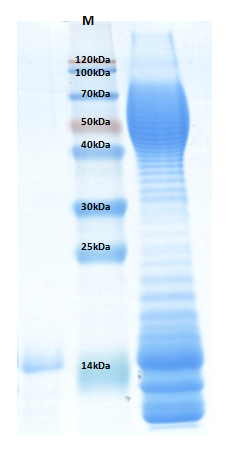


**B.**


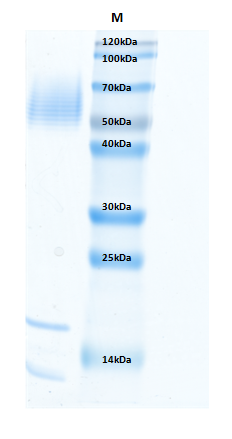

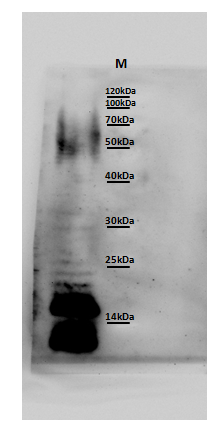

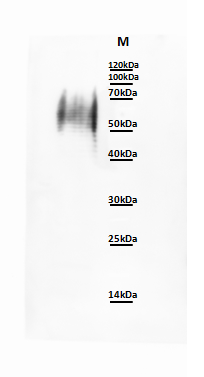


**Fig. 3A**

**
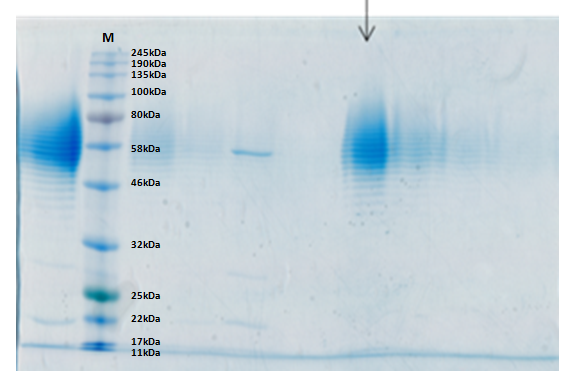
**

**Figure 4.**

**B.**


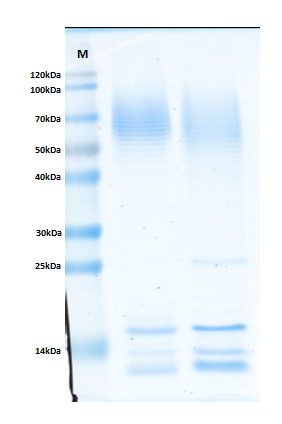

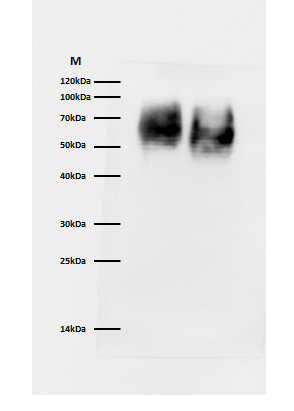


**Supplementary Fig. 1**

**
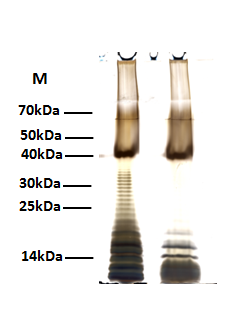
**

**Supplementary Fig. 2**

**
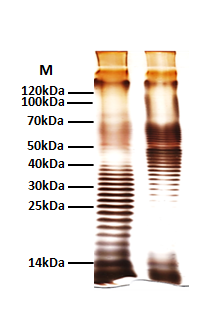
**

**Supplementary Fig. 3**

**
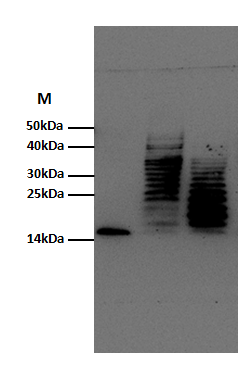
**

**Supplementary Fig. 5**


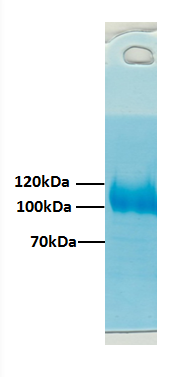

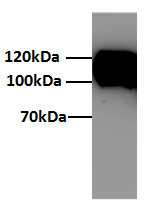

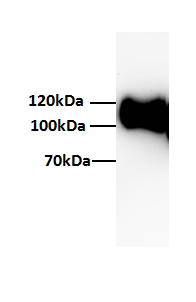


**Supplementary Fig. 7**

**
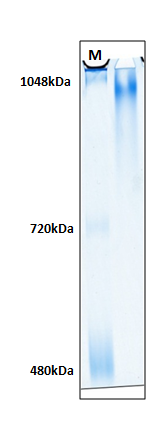
**

**Supplementary Fig. 10**


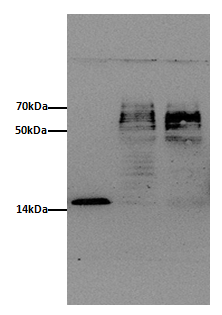

Supplement: Supplementary file 1 — Full length gels-blots [file 41541_2017_37_MOESM1_ESM.docx]
