# Supplementary material for: Design and production of conjugate vaccines against S. Paratyphi A using an O-linked glycosylation system in vivo
Source: NPJ Vaccines. 2018 Feb 5;3:4. doi: 10.1038/s41541-017-0037-1 (PMC5799188; doi:10.1038/s41541-017-0037-1)
Supplement: Supplementary file 2 — Supplemental figure legends [file 41541_2017_37_MOESM2_ESM.docx]

**Supplemental figure legends**

**Supplementary Figure 1. Confirmation of *waaL* knockout strain 50973DW.** (A) PCR assays to confirm the deletion of *waaL* in strain 50973DW using three pairs of primers (kan, out, and in) (top). The locations of the three primer pairs are indicated (bottom). (B) Silver staining of lipopolysaccharide from *S.* Paratyphi A strains CMCC 50973 and 50973DW. Samples derive from the same experiment and were processed in parallel.

**Supplementary Figure 2. Confirmation of *cld* knockout strain 50973DC.** (A) PCR assays to confirm the deletion of *cld* in strain SPA50973 using two pairs of primers: cat (left) and out (right). (B) A plasmid expressing *S*. Typhimurium gene *cld*_LT2_ was transformed into *S*. Paratyphi A strain 50973DC. Silver staining was used to observe the pattern of lipopolysaccharides in the transformant compared with wild-type strain SPA50973. Samples derive from the same experiment and were processed in parallel.

**Supplementary Figure 3.** Western blot analysis of the glycosylation of CTB4573H with or without Cld in strain 50973DW. Samples derive from the same experiment and were processed in parallel.

**Supplementary Figure 4.** Analysis of purified CTB4573H-OPS by size exclusion high-performance liquid chromatography (TSK G4000SWXL, diameter, 7.8 × 300 mm). The mobile phase consisted of 50 mM phosphate and 0.9% NaCl.

**Supplementary Figure 5.** Coomassie blue staining and western blot analysis of the purified rEPA4573H-OPS using anti-EPA antibody and *S.* Paratyphi A O2 serum. Samples derive from the same experiment and gels/blots were processed in parallel.

**Supplementary Figure 6. Evaluation of the immune effects of glycoproteins against LPS in mice.** Pre immune and at 10 days after each immunizations, serum was harvested from mice by tail clip, and the IgG titers against LPS from SPA50973 were measured. A t-test was used to compare the differences in IgG titer between the different treatment groups (***, P<0.001).

**Supplementary Figure 7.** Native-polyacrylamide gel electrophoresis was performed to identify purified CTB4573H-OPS. Samples derive from the same experiment and were processed in parallel.

**Supplementary Figure 8. Analysis of CTB4573-OPS.** (A) Proteins in the purified CTB4573-OPS sample were detected by liquid chromatography-MS/MS. * means the protein did not be detected in replicate experiments. (B) The melt curves in different pHs of purified CTB4573-OPS.

**Supplementary Figure 9. Evaluation of the immune effect of the CTB4573-OPS.** (A) 60 BALB/c mice were immunized with purified CTB4573-OPS, along with 10% Al(OH)_3_ adjuvant. Serum was harvested 10 days after the third immunizations and ELISA was performed to evaluate the titer of IgG antibody against SPA50973 LPS. (B) Complement bactericidal activity of the serum to CTB4573-OPS from panel A was tested against SPA50973 and five other Chinese *S.* Paratyphi A epidemic strains. The error bars indicate the range.

**Supplementary Figure 10.** The sequence immediately following glycosylation site 63S was changed to AP-P2 using vector pET28a-pglL-CTB4563P2H. pET28a-pglL-CTB4573H and pET28a-pglL-CTB4563P2H were separately transformed into strain 50973DWC carrying pACU184-cld_LT2_. Following induction with IPTG to co-express Cld_LT2_, PglL, and CTB4573H/CTB4563P2H, whole cell samples were separated by SDS-PAGE, and western blot analysis was performed to detect the glycosylation. A control strain expressing only Cld_LT2_ and CTB4573H was also included. Samples derive from the same experiment and were processed in parallel.
