# Supplementary figures and images for: Design and production of conjugate vaccines against S. Paratyphi A using an O-linked glycosylation system in vivo
Source: NPJ Vaccines. 2018 Feb 5;3:4. doi: 10.1038/s41541-017-0037-1 (PMC5799188; doi:10.1038/s41541-017-0037-1)

**Supplementary Fig. 1**


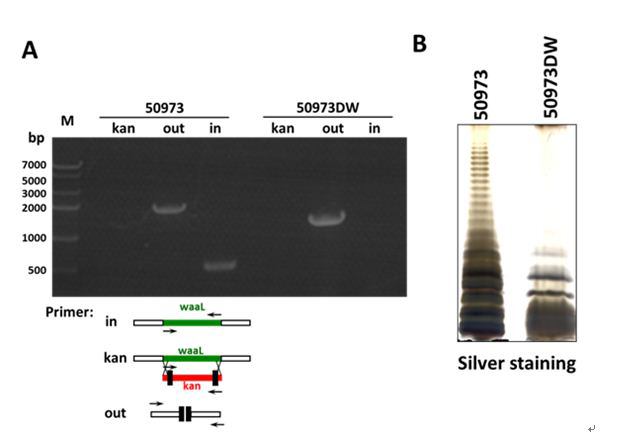

Supplement: Supplementary file 3 — Supplementary Fig. 1 [file 41541_2017_37_MOESM3_ESM.docx]

**Supplementary Fig. 2**


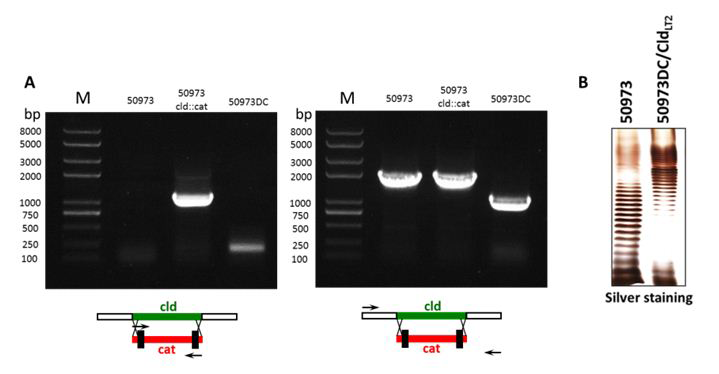

Supplement: Supplementary file 4 — Supplementary Fig. 2 [file 41541_2017_37_MOESM4_ESM.docx]

**Supplementary Fig. 3**


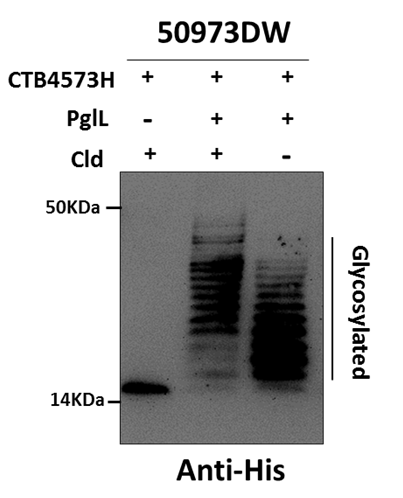

Supplement: Supplementary file 5 — Supplementary Fig. 3 [file 41541_2017_37_MOESM5_ESM.docx]

**Supplementary Fig. 4**


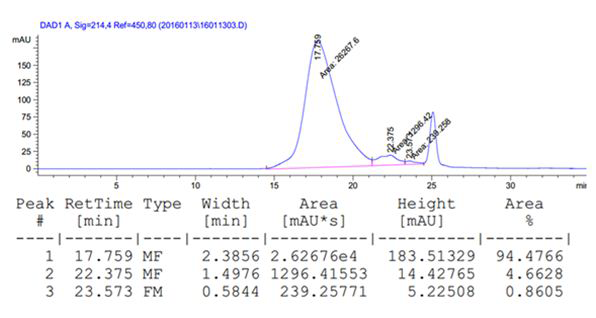

Supplement: Supplementary file 6 — Supplementary Fig. 4 [file 41541_2017_37_MOESM6_ESM.docx]

**Supplementary Fig. 5**


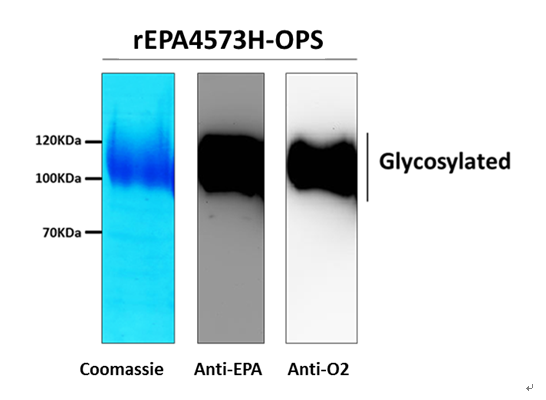

Supplement: Supplementary file 7 — Supplementary Fig. 5 [file 41541_2017_37_MOESM7_ESM.docx]

**Supplementary Fig. 6**


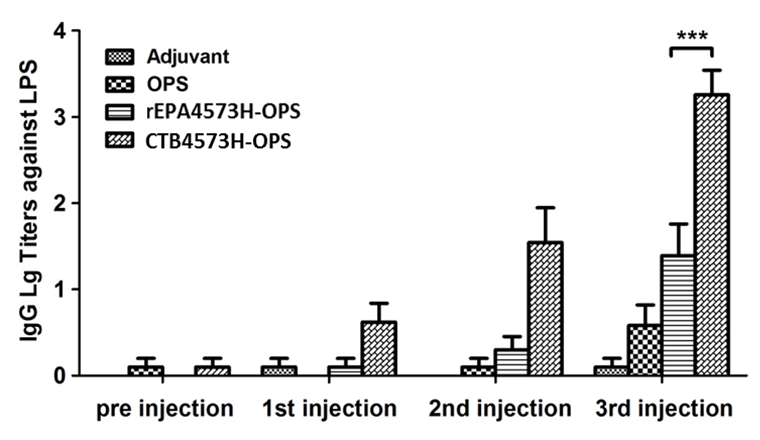

Supplement: Supplementary file 8 — Supplementary Fig. 6 [file 41541_2017_37_MOESM8_ESM.docx]

**Supplementary Fig. 7**


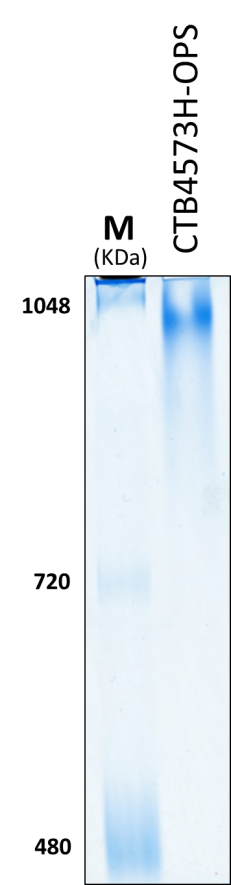

Supplement: Supplementary file 9 — Supplementary Fig. 7 [file 41541_2017_37_MOESM9_ESM.docx]

**Supplementary Fig. 8**

**
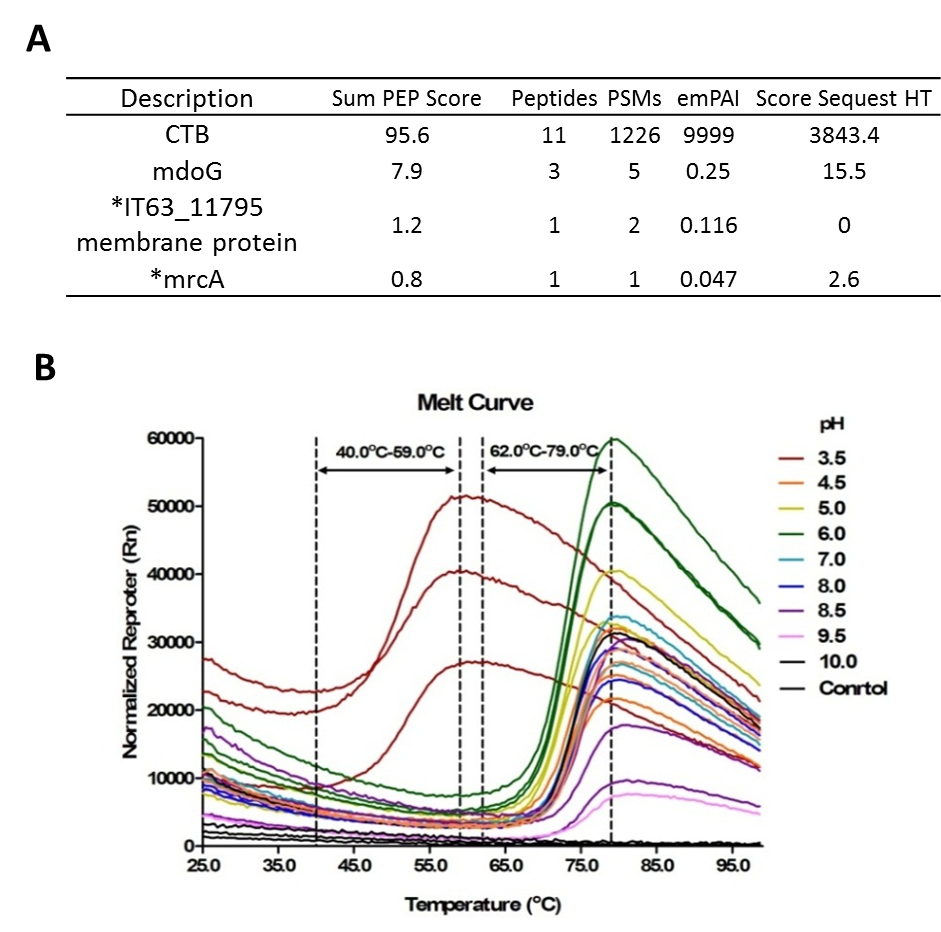
**

Supplement: Supplementary file 10 — Supplementary Fig. 8 [file 41541_2017_37_MOESM10_ESM.docx]

**Supplementary Fig. 9**


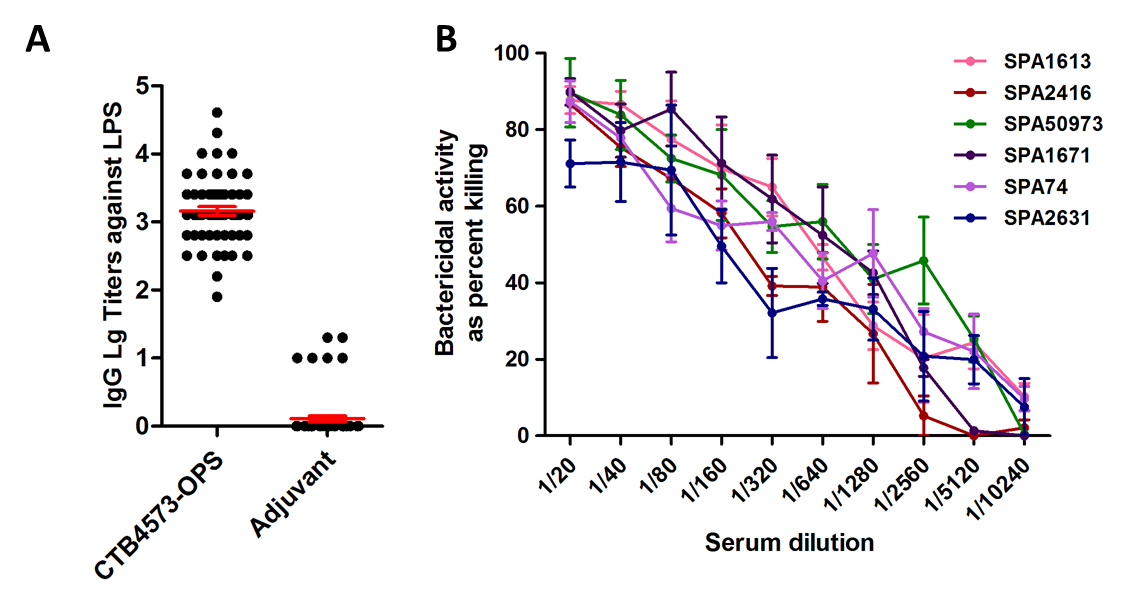

Supplement: Supplementary file 11 — Supplementary Fig. 9 [file 41541_2017_37_MOESM11_ESM.docx]

**Supplementary Fig. 10**


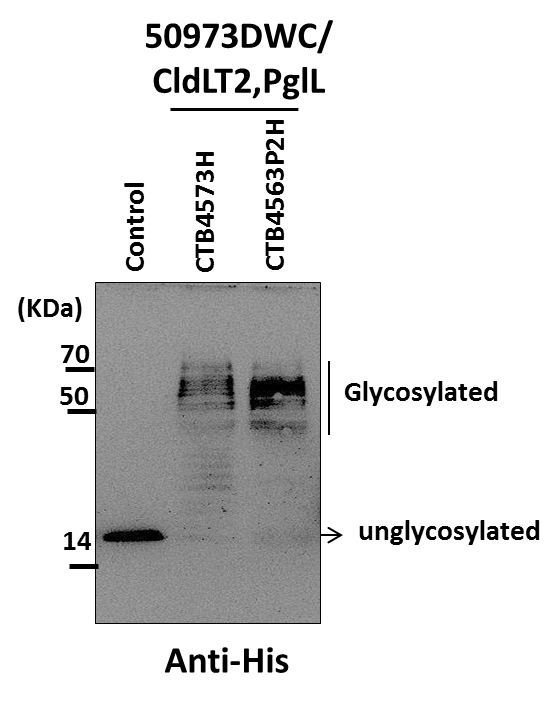

Supplement: Supplementary file 12 — Supplementary Fig. 10 [file 41541_2017_37_MOESM12_ESM.docx]
